# Tuberculosis and foreign-born populations in the United States: A mixed methods pilot study of media reporting and political identification

**DOI:** 10.1371/journal.pone.0230967

**Published:** 2020-04-21

**Authors:** Angel N. Desai, Shravanthi M. Seshasayee, Maimuna S. Majumder, Britta Lassmann, Lawrence C. Madoff, Emily L. Cohn, John S. Brownstein

**Affiliations:** 1 Division of Infectious Diseases, Brigham & Women’s Hospital, Boston, MA, United States of America; 2 International Society for Infectious Diseases, Boston, MA, United States of America; 3 Center for Outcomes Research and Evaluation, Maine Medical Center Research Institute, Portland, Maine, United States of America; 4 Computational Health Informatics Program, Boston Children’s Hospital, Boston, MA United States of America; 5 Department of Pediatrics, Harvard Medical School, Boston Children's Hospital, Boston, MA, United States of America; 6 Computational Epidemiology Lab, Boston Children's Hospital, Boston, MA, United States of America; 7 Division of Infectious Diseases, University of Massachusetts, Worcester, MA, United States of America; University of Cape Coast, GHANA

## Abstract

**Background:**

Media reporting on communicable diseases has been demonstrated to affect the perception of the public. Communicable disease reporting related to foreign-born persons has not yet been evaluated.

**Objective:**

Examine how political leaning in the media affects reporting on tuberculosis (TB) in foreign-born persons.

**Methods:**

HealthMap, a digital surveillance platform that aggregates news sources on global infectious diseases, was used. Data was queried for media reports from the U.S. between 2011–2019, containing the term “TB” or “tuberculosis” and “foreign born”, “refugee (s),” or “im (migrants).” Reports were reviewed to exclude duplicates and non-human cases. Each media source was rated using two independent media bias indicators to assess political leaning. Forty-six non-tuberculosis reports were randomly sampled and evaluated as a control. Two independent reviewers performed sentiment analysis on each report.

**Results:**

Of 891 TB-associated reports in the US, 46 referenced foreign-born individuals, and were included in this analysis. 60.9% (28) of reports were published in right-leaning news media and 6.5% (3) of reports in left-leaning media, while 39.1% (18) of the control group reports were published in left- leaning media and 10.9% (5) in right-leaning media (p < .001). 43% (20) of all study reports were posted in 2016. Sentiment analysis revealed that right-leaning reports often portrayed foreign-born persons negatively.

**Conclusion:**

Preliminary data from this pilot suggest that political leaning may affect reporting on TB in US foreign-born populations. Right-leaning news organizations produced the most reports on TB, and the majority of these reports portrayed foreign-born persons negatively. In addition, the control group comprised of non-TB, non-foreign born reports on communicable diseases featured a higher percentage of left-leaning news outlets, suggesting that reporting on TB in foreign-born individuals may be of greater interest to right-leaning outlets. Further investigation both in the U.S. and globally is needed.

## Introduction

Mass media has the ability to impact public perceptions of medicine and disease processes [1]. Given its wide scope and the increasing availability of media through the digitalization of the popular press, online news reports have become an important source for public health care information [2]. This is especially significant in the reporting of communicable diseases, where positive behavior change–as well as desensitization or even stigmatization–in the setting of an epidemic can occur [3,4].

Concurrently, media coverage of immigration in the United States has become increasingly prevalent in the setting of political polarization as well as the rise of “new media” [5]. Recent studies have found that the American public has become ideologically consistent and partisan [6]. This finding is also reflected in media consumption patterns based on self-identified placement along the ideological spectrum [6].

Prior studies have examined the relationships between news reporting, public perception, and communicable diseases [7–10]. However, none to our knowledge have investigated how political ideology may influence news content as it pertains to foreign-born individuals. The aim of this pilot study was to examine the relationship between communicable diseases and foreign-born persons as portrayed by the mass media. Tuberculosis (TB) was chosen as an index infectious pathogen as it is an important public health issue that is often linked to foreign-born persons in the United States [11]. Although the incidence of TB is falling in the United States, 70.1% of all reported TB cases in the United States in 2017 occurred among non-U.S.-born persons [11,12]. The majority of these cases are due to reactivation of latent TB; however factors such as active overseas screening, improved surveillance, declining morbidity, and changes in demographics of recent entrants may be contributing to overall decreasing incidence of TB [13, 14]. Despite this, TB remains a highly stigmatized and often misunderstood disease and was chosen for this pilot study due to these criteria.

## Methods

### Data sources

In order to evaluate media content relating foreign-born individuals to TB, HealthMap–an internet-based communicable disease surveillance tool was used. HealthMap was founded in 2006 and uses online, informal data sources to provide continuous disease outbreak monitoring [15, 16]. It aggregates disparate news sources, official reports, and expert-curated discussion for this purpose. For this study, the HealthMap search engine was queried for news media reports pertaining to U.S. outbreaks that contained the term “TB” or “tuberculosis” and “foreign born”, “refugee(s)” or “im (migrants)” between January 2011 to June 2019. Only English language reports were searched and included. Duplicate records, non-human cases, and reports without active URLs were excluded. Of note, if one event was reported by multiple media agencies, all relevant reports were retained in order to compare reporting on TB and foreign-born persons between various media outlets. Analysts reviewed auto-populated fields and amended for style accordingly.

### Data extraction

**Fig 1:**
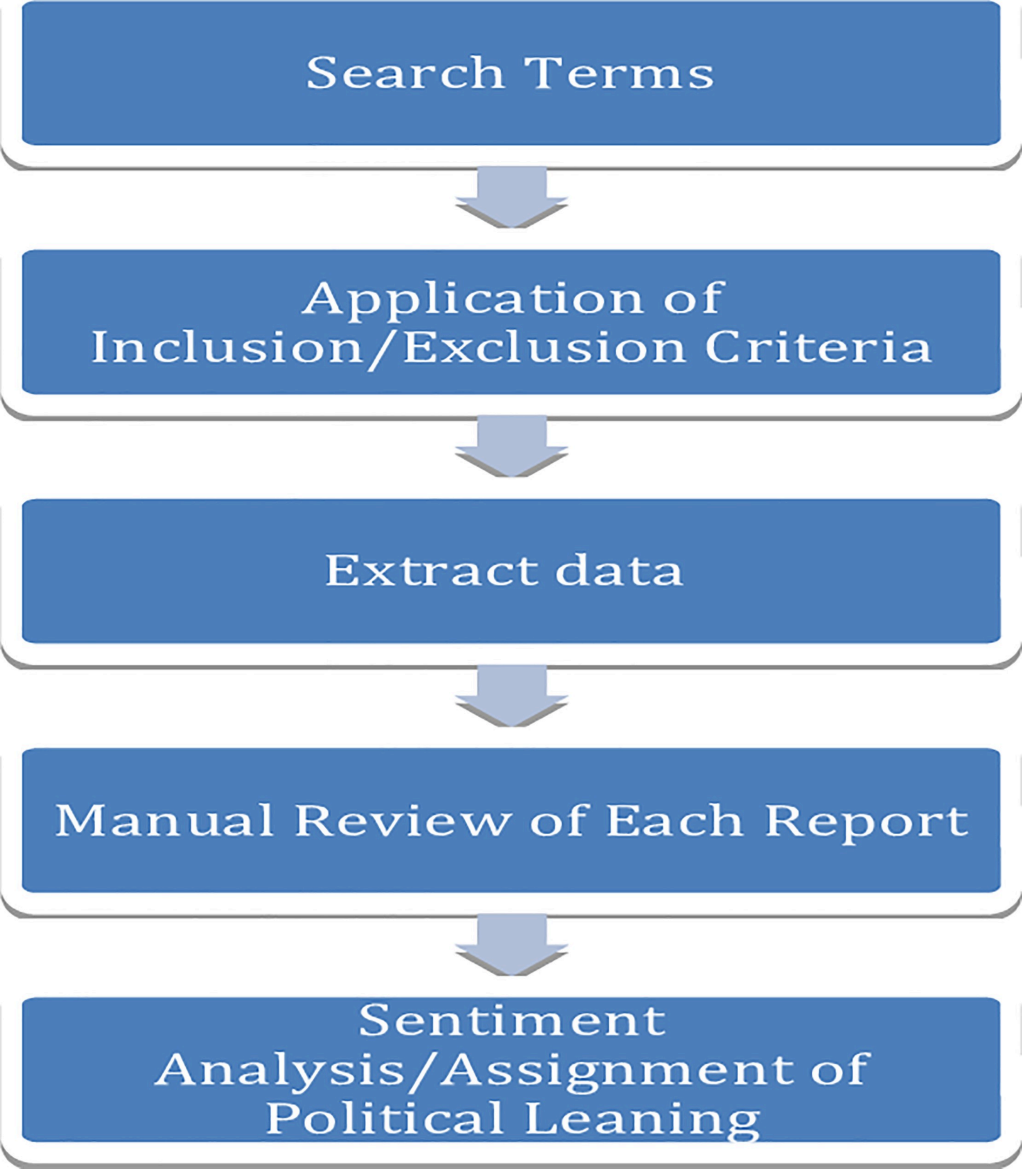
Report collection and data entry.

Each report was manually reviewed by two independent investigators (AND and SMS). Data on media source, date of publication, and confirmed case counts (i.e., disease incidence as reported by regional health departments) were extracted where available (Fig 1). Media sources for each report were rated by utilizing two different media bias indicators: *MediaBiasFactCheck*.*com* was the initial rating system used. If political leaning was unavailable, a second indicator, *AllSides*.*com*, was applied [17,18]. Both media bias indicators employ rating systems that are further described elsewhere in an attempt to categorize potential political leaning [17,18]. Ratings for each media source pertaining to an individual news report were delineated along a spectrum characterizing political leaning, ranging from right, center, to left. Using *MediaBiasFactCheck*.*com* and *AllSides*.*com* definitions, right-leaning political bias was characterized as adhering to “conservative” values and philosophies whereas left-leaning political bias was defined as “liberal” values and political thought. Pro-science was used to indicate factual or evidence-based reports, and center indicated that no obvious right- or left-leaning statements were present. For presentation purposes, pro-science and center reports were categorized together in this analysis. This is in line with the spectrum of political ideology as generally classified in the United States popular media [19]. For the purposes of this analysis, left and left-center bias was characterized as left-leaning, and right and right-center bias were characterized as right-leaning. Media outlets for which political bias could not be ascertained from either bias indicator were retained in the final analysis and characterized as “unknown.” Internet-based, topic-specific news aggregators included in the HealthMap dataset were rated as center-leaning given that the purpose of these websites is to aggregate content from all online news sources.

A random number generator was used to extract non-TB, non-foreign-born associated communicable disease reports from the HealthMap search engine to act as a control. A select group of other communicable infections as listed below were the primary sample used as a control to match perception of contagiousness and severity of TB in order to minimize confounding. Other communicable infections were defined as the following: Cryptococcus, cryptosporidium, cytomegalovirus, herpes simplex, pneumonia, pneumococcal pneumonia, salmonella, and toxoplasmosis.

### Sentiment analysis

Two reviewers (AND and SMS) systematically rated online news reports retained in the final study sample to qualitatively assess sentiment (e.g., language and tone, also referred to as “affect”) in the headlines and text of each report. Affect ratings were categorized as positive, negative, or neutral. Categories were defined *a priori* as follows: A report was rated as possessing a negative affect if misleading or inflated disease burdens were presented, if negative remarks were made regarding foreign-born populations, or if a call for more stringent policies on immigration, asylum seekers, or provision of public services to foreign-born populations was made. A report was considered to have a positive affect if there was mention of or a recommendation for public health improvements. Reports were determined to be neutral if they provided information on the incidence or prevalence of the disease, with no further recommendations or calls for action. Inter-rater reliability in sentiment affect classification was determined using Cohen’s κ, following initial calibration in which each reviewer independently rated a sample of 20 reports (10 each of the study and control datasets). In the final analysis, cases where a disagreement between reviewers regarding sentiment classification of a particular report occurred, discussion and reconciliation took place between reviewers to identify a unified rating. Incidence and prevalence data presented in each news report were independently verified against official data sources including the U.S. Centers for Disease Control and regional public health departments when applicable. The study analyzed publicly available data and no individual human subjects were involved.

### Data analysis

Quantitative analysis using descriptive statistics was conducted utilizing Microsoft Excel^®^ and Stata (version 13.1; StataCorp LP). A χ2-squared test of independence was performed to assess differences in political leaning among the media sources that reported on study cases versus controls. An alpha level of less than or equal to .05 was considered statistically significant.

## Results

**Fig 2:**
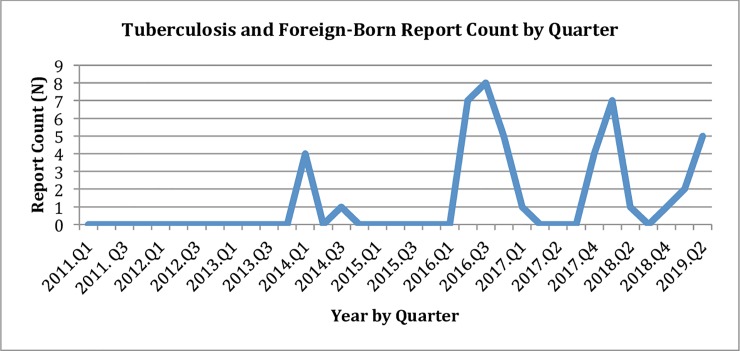
Tuberculosis and foreign-born persons report count by quarter.

From January 1, 2011, to January 1, 2019, there were 5.633 million alerts collected by the HealthMap system. 891 reports involving the United States and tuberculosis were extracted from the HealthMap database during this time period. Forty-six of these reports involved foreign-born individuals. Forty-six reports obtained from HealthMap were also extracted simultaneously to act as a control group. No reports involving TB and foreign-born individuals were published from 2011 to 2013. From 2014 to 2019, an average of five reports involving tuberculosis and foreign-born individuals were published per year. 43% (20) of all media reports evaluated over the course of this pilot study were published in 2016 (Fig 2). Three reports were extracted from non-U.S. or U.S. subsidiary outlets. These were retained and included in the final cohort as they pertained to U.S. TB outbreaks, and no additional reports extracted from the initial search that pertained to TB and foreign-born persons belonged to non-U.S. media sources.

**Fig 3:**
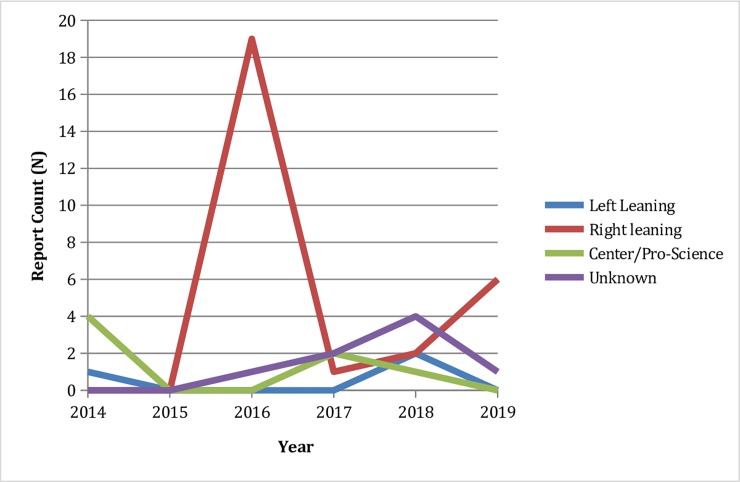
TB report count categorized by political leaning by year.

Media sources corresponding to each of the news reports were individually categorized by political leaning using *MediaBiasFactCheck*.*com* and *AllSides*.*com*. No disagreements occurred between media bias indicators. Table 1 shows the report count categorized by political leaning for TB and foreign-born reports as well as for the control group. Compared to their control counterparts, media sources reporting on tuberculosis among foreign-born populations in the United States were found to be more likely to be right-leaning (60.9% vs. 10.9%), and less likely to be center/pro-science (6.5% vs. 39.1%) or left-leaning (15.2% vs. 34.8%). A x2 test of independence demonstrated that reporting on TB and foreign-born populations in the US was associated with media political leaning (p < .001). Fig 3 demonstrates political leaning for TB and foreign-born reports by year.

**Table 1:** Report count categorized by political leaning.

	***TB & Foreign-Born Reports*, *N (%)***	***Controls*, *N (%)***
*Left-leaning*	3 (6.5)	18 (39.1)
*Right-leaning*	28 (60.9)	5 (10.9)
*Center/Pro-Science*	7 (15.2)	16 (34.8)
*Unknown*	8 (17.4)	7 (15.2)
*Total*	46 (100)	46 (100)

Sentiment analysis was conducted on each individual news report to evaluate whether the content of reporting reflected a positive, negative, or neutral tone towards foreign-born populations specifically. Table 2 demonstrates the distribution of positive, negative, and neutral sentiment per report reviewed. Table 3 further delineates sentiment distribution with the headline for each article assessed and inter-rater reliability. Inter-rater agreement in sentiment classification based on calibration (Cohen’s k = 0.87) was high. This analysis demonstrated that the majority of right-leaning reports were associated with a negative affect, while no right-leaning reports were associated with a positive affect. Reports that reflected a negative affect often discussed latent and active TB interchangeably. For example, one report classified as negative and originating from a right-leaning news outlet noted that “…[a] significant percentage of resettled refugees never complete their health screenings, so they may be wandering around untreated, for any of a number of diseases including tuberculosis…” while referencing latent TB cases [20]. Negative reports also discussed TB and foreign-born persons in the context of policy and politics. One such report noted, “[t]he government’s increased inflow of tuberculosis-carrying migrants appears to have reversed a 23-year decline of contagious tuberculosis cases inside the United States. The jump in foreign-born cases from 22 percent in 1986 to 66 percent in 2015 is caused by the federal government’s policy of accepting more tuberculosis-infected migrants from countries with large-scale contagions of the deadly and debilitating disease [21].”

**Table 2:** Sentiment categorization by affect and political leaning.

S	Positive Affect	Negative Affect	Neutral Affect	Total
Left-leaning/Liberal	1	1	1	3
Right-leaning/Conservative	0	22	6	28
Center/Pro-Science	1	0	6	7
Unknown	0	0	8	8
Total	2	23	21	46

**Table 3:** Sentiment categorization by article and rater.

Headline	Rater #1	Rater #2
2 cases of active tuberculosis found in Central American immigrants brought to El Paso for case processing	Negative	Negative
Houston efforts key to ending tuberculosis in US	Positive	Positive
US—Colorado: Tuberculosis case at Aurora ICE facility	Neutral	Neutral
22 Percent of Resettled Refugees in Minnesota Test Positive for Tuberculosis	Negative	Negative
Arizona: Most of State's 222 Active TB Cases Among Refugees 'Caused by Latent Tuberculosis Infections'	Negative	Negative
Workers at 2 Minnesota Hospitals Diagnosed with Active TB	Negative	Negative
US Tuberculosis Cases Rise as Foreign-Born Patients Triple 1986 Caseload Percentage	Negative	Negative
Refugees double TB cases in Texas	Negative	Negative
Vermont Health Department concealing number of refugees with contagious TB	Neutral*	Negative
Seven refugees in Idaho 'have been diagnosed with active tuberculosis'	Negative	Negative
Three Refugees Diagnosed With Active TB in Vermont Over Past Seven Months	Negative	Negative
Vermont Admits Seventeen Refugees Diagnosed With Active TB	Negative	Negative
Six Refugees in Wisconsin Diagnosed with Multi-Drug Resistant TB Since 2014	Negative	Negative
Three Foreign-Born Cases of Multi-Drug Resistant TB in Nashville, Tennessee	Negative	Negative
Eleven Refugees Diagnosed With Active Tuberculosis Around Akron, Ohio	Negative	Negative
Five Foreign-Born Cases of Multi-Drug Resistant TB Diagnosed in Philadelphia	Negative	Negative
Twenty-One Refugees Diagnosed with Active TB in Nebraska	Negative	Negative
Tuberculosis Surfaces at Texas High School	Neutral	Neutral
296 Refugees Diagnosed with Active TB in Minnesota, Ten Times Any Other State; Majority Are Somalis	Negative	Negative
MN Refugee TB Infections Are Ten Times Higher Than Other States	Negative	Negative
Ingham County, Michigan: One Refugee Diagnosed With Multi-Drug Resistant TB; 22 Percent Have Latent TB	Negative	Negative
Illinois Confirms Eight Refugees Diagnosed with Active TB Upon Arrival	Neutral*	Negative
Another Case of Active TB Diagnosed in Hennepin County, Minnesota Schools	Negative	Negative
More than 9000 People in the US Got Tuberculosis Last Year. Who Were They?	Neutral	Neutral
Panic as TB is on the rise in New York for the first time since the 1990s	Neutral	Neutral
Fargo City Commissioner accused Lutheran Social Services of hiding tuberculosis risk; makes motion to stop refugee inflow into Fargo	Neutral	Neutral
“Crisis” of Seriously Ill Migrants Slams Border Patrol-TB, Pneumonia, Influenza	Negative	Negative
Government Releasing Sick Illegals in American Communities	Negative	Negative
Pennsylvania Congressman John Joyce takes back claims of tuberculosis-infected migrants crossing border	Neutral	Neutral
Border Patrol Official: Central Americans Entering US With Contagious Health Conditions	Negative	Negative
While Republicans visited Yuma, a bogus tuberculosis scare on the border	Neutral	Neutral
PRO/EDR> Tuberculosis, MDR—USA: (MN) fatal, Hmong, senior center	Neutral	Neutral
PRO/AH/EDR> Tuberculosis—USA: children, foreign-born parents	Neutral	Neutral
Tuberculosis Sees Biggest Increase In NYC Since 1992	Neutral	Neutral
Latent TB testing, treatment cost-effective for non-US born residents in most cases	Neutral	Neutral
Trends in Tuberculosis-United States, 2013	Neutral	Neutral
NYC Tuberculosis Cases Among Immigrants Continue to Rise	Neutral	Neutral
Implementation of New TB Screening Requirements for U.S.-Bound Immigrants and Refugees-2007-2014	Positive	Positive
Older Hmong Residents Affected by Tuberculosis Outbreak	Neutral	Neutral
Six have died from multi-drug-resistant tuberculosis in Ramsey County, officials say	Neutral	Neutral
Whole-Genome Sequencing Identifies Multidrug-Resistant Tuberculosis Among Refugees	Neutral	Neutral
2 cases of multi-drug resistant TB confirmed in Northeast Ga.	Neutral	Neutral
Why is tuberculosis on the rise in NYC but not New Jersey?	Neutral	Neutral
WBGO Investigates: NYC TB Case Spike Reverses Years of Progress	Positive	Positive
Are Eradicated Diseases Making a Comeback Because of Immigrants …	Neutral	Neutral
NYC TB Case Spike Reverses Years of Progress	Positive	Positive

*Rater sentiment reconciled to negative

Blue: Left-leaning/Liberal

Orange: Right-leaning/Conservative

Green: Center/Pro-Science

Yellow: Unknown

Accuracy of reporting content was verified by comparing case counts with surveillance data available through the U.S Centers for Disease Control [22]. In addition, regional public health department data was used, as this information is available on a county or health district level in the United States and aggregated by HealthMap [23]. In 5 reports, individual level outbreak data could not be validated, however annual prevalence estimates for a state or region that were also presented in these reports were verified. Case counts were accurate for all reports curated in this study.

## Discussion

Communicable disease reporting has a unique and burgeoning relationship with online news outlets. This study examined the potential for bias based on political ideology in the reporting of the incidence and prevalence of tuberculosis among foreign-born persons in the United States, and potential for bias along ideological spectrums reflected in online news reports curated through the HealthMap digital disease detection and media monitoring system. This study suggests that right-leaning media sources tended to report more frequently on tuberculosis cases in foreign-born persons as compared to left-leaning media outlets, and that those reports tended to reflect negative sentiment as well.

This is also notable as the incidence of tuberculosis in the United States overall has been declining over the past several years [11]. Conversely, in a sample of communicable disease reporting from HealthMap overall, the frequency of reporting was more heavily represented in left-leaning media outlets. While these findings should be interpreted with caution, this may suggest that reporting on TB in foreign-born persons may be unequally distributed along the political spectrum at baseline. Right-leaning media outlets may report on TB in foreign-born persons more frequently than left-leaning media, and left-leaning media outlets may report on other communicable diseases more frequently, perhaps highlighting a difference in reporting priorities, interest, and political will. Prior studies investigating media and communicable diseases have suggested that discordance between frequency of print and online reporting and actual risk as defined by mortality rates may exist [24,25]. Moreover, media reports tend to amplify events that are perceived to be rare or dramatic, and the influence of the mass media has been studied in the context of U.S. political agenda setting [1, 26].

The mean number of reports from 2014 to 2019 was 5 reports per year. The relatively few news pieces may reflect the focused nature of this analysis given that only reports curated from HealthMap on tuberculosis in foreign-born individuals in the United States were included. These results may also reflect the intended focus of digital disease detection tools on clusters or outbreak events that are unusual or more numerous than expected. As such, events involving “chronic” infectious diseases such as tuberculosis are not always captured on HealthMap [15]. Despite this, it is notable that 43% (20) of all reports noted in this pilot study were posted in 2016. The political environment during the summer of 2016 in the United States and rhetoric surrounding immigration may have contributed to the concurrent increase in reporting. Conversely, media priorities may have also affected political discourse. One study by McCombs et al. noted that a strong relationship exists between the issues the media emphasizes during an election cycle and voter judgment [27]. In addition, Menjivar noted that negative media portrayals of immigrants could contribute to hostilities at the level of public discourse [28]. Given the small number of reports curated for this pilot and the observational nature of the study, a correlation between political events that may have influenced increased reporting and the number of reports occurring in 2016 could not be assessed. However it may be worth considering the effects of media sentiment in future studies.

This is the first study to our knowledge that has examined the association between communicable disease reporting, media bias, and foreign-born persons. Causal links, however, may be difficult to establish. Studies investigating media bias and refugee populations have demonstrated that news reporting does, on occasion, target mass consumer appeal as opposed to reflecting structural or real-time certainties [29]. Conversely, the popular press has also shed light on ongoing humanitarian crises. The relationship between the media and immigration remains complex at best and changing national politics and belief systems about migrants may be both an influence on as well as influenced by the mass media.

This study had several limitations. Despite the relative strengths of the surveillance system used to generate reports included in this study, it was limited by the small sample size that met the inclusion criteria. Given that this is a pilot study, any conclusions drawn here should be interpreted judiciously. Additional studies involving a larger sample size should be conducted in the future. Only news reports written in English were included for the purposes of this study, limited both by geographic scope of the study as well as accuracy and validity of media bias indicators. Both media bias indicators attempt to apply rigorous quality metrics to establish accuracy, however bias is ultimately a subjective parameter and tools that attempt to classify bias should be interpreted cautiously. It is possible that the entirety of news reports addressing tuberculosis and foreign-born persons in the United States has not been captured here, particularly given that internet-based disease monitoring and evaluation tools such as HealthMap focus on unusual events of significance. While tuberculosis remains relatively rare in the United States, those events that do occur may not have produced signals significant enough for reporting on HealthMap. In addition, HealthMap reports undergo an initial process of de-duplication prior to posting, and as such, some reports on the same outbreak from different news sources may have been removed prior to inclusion for this study. While the control group was chosen in an attempt to balance TB, an often-stigmatized disease, against other communicable diseases, a true control is difficult to define, and may not be optimal in this setting. Finally, some reports were unable to be categorized by political leaning, as they could not be identified in either media bias indicator. While the reports were retained for the purpose of analysis, it is possible that unknown reports *do* have a political leaning that could have affected the results. However, the total number of unknown reports remained small, and if conservative estimates were applied and these reports were added to either left- or right-leaning categories, the overall balance of media biases would not have changed enough to significantly alter our results.

## Conclusion

Encouraging public discourse on communicable diseases is critical for the dissemination of vital health information. Equally important however, is the promotion of accurate and unbiased health reporting, particularly when involving vulnerable populations. This pilot study suggests that political leaning may affect reporting on foreign-born populations and tuberculosis, however additional studies examining a larger sample size and diversity of communicable disease states are needed.
